# Implementation of tobacco control measures in the Gulf Cooperation Council countries, 2008–2020

**DOI:** 10.1186/s13011-021-00393-8

**Published:** 2021-07-03

**Authors:** Sarah S. Monshi, Jennifer Ibrahim

**Affiliations:** 1 Department of Health Services Administration and Policy, College of Public Health, Temple University, 1301 Cecil B. Moore Avenue, Philadelphia, PA 19122 USA; 2 Department of Health Services Administration, Umm Al-Qura University, Mecca, Saudi Arabia

**Keywords:** Tobacco control measures, Anti-tobacco policy, Smoking, Tobacco use, Gulf Cooperation Council, Framework convention on tobacco control

## Abstract

**Background:**

The World Health Organization (WHO) Framework Convention on Tobacco Control (FCTC) was developed to assist nations in reducing the demand and supply of tobacco. As of 2020, 182 nations joined the FCTC, agreeing to implement the recommended tobacco control measures. The Gulf Cooperation Council (GCC) countries, including Bahrain, Kuwait, Oman, Qatar, Saudi Arabia, and United Arab Emirates (UAE) ratified the WHO FCTC by August 2006. Given the unique political, cultural, and religious context – and known tobacco industry efforts to influence tobacco use- in these nations, a careful examination of the translation of FCTC measures into policy is needed. This study aimed to assess the implementation of FCTC tobacco control measures at the national level within the six GCC countries.

**Method:**

We collected and coded the FCTC measures that were implemented in the GCC countries. We examined trends and variations of the implementation between 2008 and 2020.

**Results:**

GCC countries implemented most FCTC measures targeting the demand for and supply of tobacco, with some variation among countries. Bahrain and Qatar were more comprehensively implementing FCTC measures while Kuwait and Oman implemented the least number of the FCTC measures. Implementing measures related to tobacco prices and eliminating the illicit tobacco trade has slowly progressed in GCC countries. All GCC countries entirely banned smoking in workplaces while three countries implemented a partial ban in restaurants. Only Oman has restrictions on tobacco ads shown in media. There is progress in implementing FCTC measures related to tobacco packaging, cessation, and sale to minors in most GCC countries.

**Conclusions:**

Given the influence of the tobacco industry in the Gulf region, the findings suggest a need for ongoing surveillance to monitor the proliferation of tobacco control measures and evaluate their effectiveness. Efforts required to address tobacco use should correspond to the unique political and cultural background of the GCC countries.

## Background

### Global tobacco use

Tobacco use is responsible for more than eight million worldwide deaths annually [1] and is the greatest risk factor for many preventable diseases, including cancers and cardiovascular diseases. The harmful effect of tobacco use spreads to non-tobacco users exposed to secondhand smoke (SHS), causing preventable diseases among non-tobacco users. Globally, there are more than one million premature deaths per year due to SHS-related disease; among them 65,000 are children [1]. The global economic cost of tobacco use in 2016 accounted for $1.4 trillion [2], including costs associated with premature death, treating chronic diseases, and loss of workplace productivity [3].

### Tobacco use in the Gulf Cooperation Council countries

Despite significant efforts to control tobacco use worldwide [4], one area of the globe where tobacco use continues to be a significant problem is in the Middle East. The Gulf Cooperation Council (GCC) is a political and economic union of six countries: Bahrain, Kuwait, Oman, Qatar, Saudi Arabia, and the United Arab Emirates (UAE) located in the Middle East. They share similar economic and social characteristics (Table 1) [5]. They experienced similar economic growth due to oil discovery during the 1930 s [6]. The region is the origin of the Islamic religion, which strongly impacts the societies’ values. Islam does not clearly state that tobacco use is forbidden. Instead, it encourages individuals to preserve their overall health [7].

**Table 1:** Key characteristic of GCC countries

	Bahrain	Kuwait	Oman	Qatar	Saudi Arabia	UAE
Geographic Size (Thousand square km)	0.77	17.82	309.5	11.6	2000	71.02
Capital	Manama	Kuwait	Muscat	Doha	Riyadh	Abu Dhabi
Primary Language	Arabic	Arabic	Arabic	Arabic	Arabic	Arabic
Population (Million People)	1.37	3.97	4.16	2.44	31.52	8.26
Gross Domestic Product (Billion US Dollar)	31.13	114	69.8	164.64	646	370.3
Per Capita Gross Domestic (Thousand US Dollar)	22.71	28.7	16.8	67.5	20.8	48.33

Source: Secretariat General of the Cooperation Council for the Arab States of the Gulf [5]

**Fig. 1:**
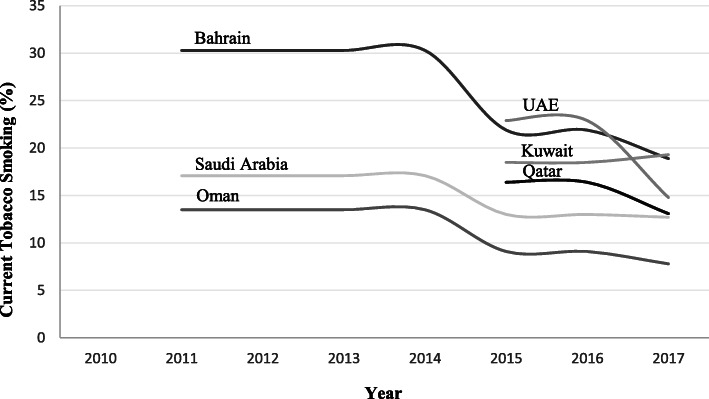
Shows age-standardized estimated prevalence of current tobacco smoking among adults (Age ≥ 15) who smoke tobacco daily and occasional in GCC countries [9]

The estimated prevalence of tobacco smoking among adults in 2017 ranged from 19.3 % in Kuwait to 7.8 % in Oman, with a higher prevalence among males than females (Fig. 1) [8, 9]. The economic burden of smoking and SHS in GCC countries accounted for $34.5 billion in 2016 [10]. The projected trend of adult tobacco use in GCC countries indicates a steady increase in tobacco use among adult males compared to females [11]. Moreover, the trend of susceptibility to initiating tobacco use among youth, aged 13 to 15 years, has increased in multiple GCC countries including Oman, Qatar, Saudi Arabia, and UAE, with a growing number of female youths who were willing to initiate tobacco use [12].

### Framework convention on tobacco control

In response to the growing tobacco use, the World Health Organization (WHO) introduced the Framework Convention on Tobacco Control (FCTC) in 2003. The FCTC is an international treaty to assist nations in overcoming the negative consequences of tobacco use through evidence-based measures intended to reduce the global demand and supply of tobacco (Table 2) [13]. The FCTC measures targeting the demand for tobacco, such as increasing tobacco taxes, protecting people from SHS, and raising public awareness against the harmful effects of tobacco. In contrast, the FCTC measures targeting tobacco supply include eliminating illicit tobacco trade and restricting the sale of tobacco to minors. As of 2020, 182 countries have joined the FCTC, agreeing to work towards implementing the recommended tobacco control measures [14].

**Table 2:** Measures from the Framework Convention on Tobacco Control

Tobacco Demand Reduction Measures	Tobacco Supply Reduction Measures
• Price and tax on tobacco • Protection from exposure to tobacco smoke • Regulation of tobacco contents • Regulation of tobacco disclosures • Packaging and labeling of tobacco products • Education, communication, training, and public awareness • Tobacco advertisement, promotion, and sponsorship • Tobacco dependence and cessation	• Illicit trade in tobacco products • Sales of tobacco to minors • Provision of support for economically viable alternative activities

Source: World Health Organization [13]

In 2008, building on the framework provided in the FCTC, the WHO developed the “MPOWER” policy package with specific measures on tobacco’s demand side. MPOWER stands for (M) monitor tobacco use, (P) protect from exposure to SHS, (O) offer help to quit tobacco, (W) warn about the danger of tobacco, (E) enforce ban on advertisement, sponsorship, and promotion, and (R) raise tobacco tax. Four years after the creation of MPOWER, the WHO introduced the protocol to eliminate illicit tobacco trade as an international treaty based on FCTC Article 15, which includes measures to regulate the manufacturing, shipping, sale, and distribution of tobacco products. Article 15 entered into force in September 2018 [15, 16].

Evidence shows that the implementation of FCTC measures leads to promising outcomes [17]. One of the key aspects of FCTC measures is changing the physical environment that influences psychosocial and behavioral factors associated with tobacco use [18, 19]. Implementing FCTC measures such as raising tobacco taxes and public awareness affects attitudes and perceptions about tobacco use, while a smoke-free policy protects individuals from exposure to SHS and creates anti-social norms toward tobacco use [18]. Other FCTC measures such as banning tobacco ads and regulating tobacco sales and trades prevent tobacco industry interference and limit the industry’s activities aimed at increasing tobacco sales [20]. Consequently, the comprehensive implementation of FCTC measures showed a reduction in tobacco consumption, preventing initiation of tobacco use, increasing quit attempts, and improving knowledge about tobacco’s harmful use [17, 21–23]. While there is remarkable evidence that supports the effectiveness of FCTC, gaps remain in implementing FCTC measures, especially those targeting the tobacco industry interferences [17]. Lack of resources and political commitment limits the evolution of tobacco control measures [24]. The implementation of FCTC measures is still in its early stages in several nations, and little is known about their impacts on populations [17, 25].

### FCTC adoption by GCC countries

**Fig. 2:**
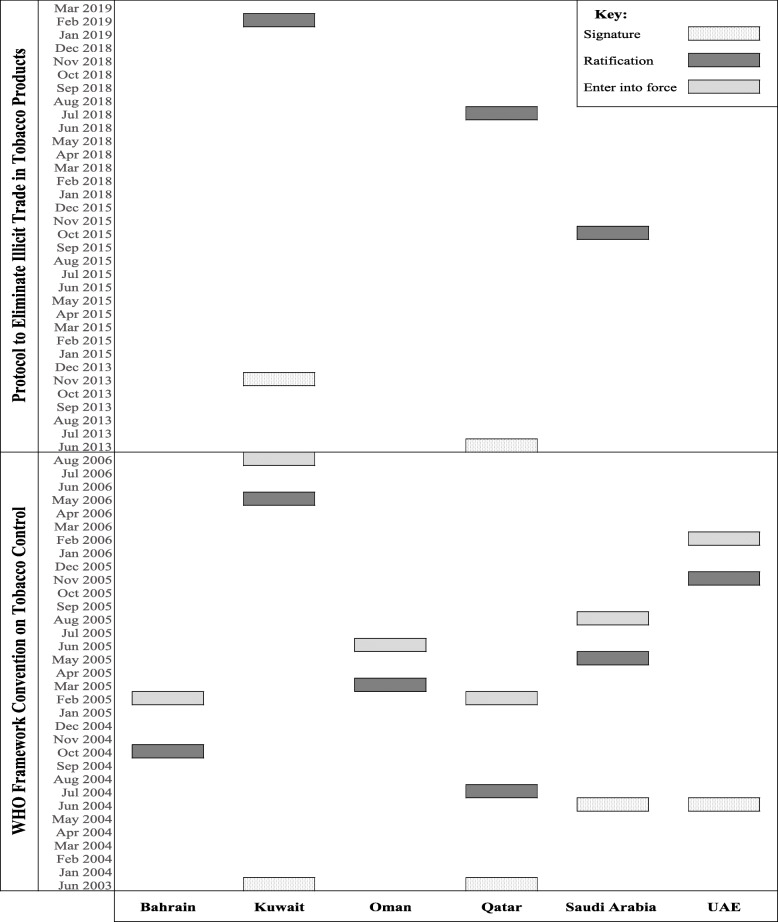
Indicates times when FCTC treaty was signed, ratified, and entered into force in GCC countries [14, 16]

Since 1994, GCC countries started to propose tobacco control laws [26]. The six GCC countries signed and ratified the FCTC by August 2006. Only three GCC countries (Kuwait, Qatar, and Saudi Arabia) ratified the protocol of eliminating illicit trade in tobacco products (Fig. 2) [14, 16]. However, the literature shows a gap in reporting the progress of FCTC implementation. A particular limitation in the existing literature is that most studies reported the implementation of MPOWER measures, which target tobacco’s demand-side [21, 26–28]. FCTC supply-reduction measures, including eliminating illicit trade in tobacco products and regulating tobacco sales to minors, were reported in much less detail in the literature.

Multiple studies have comprehensively assessed the implementation of FCTC in Africa and the Southeast Asia regions [29, 30], but there are no studies that report specifically on the FCTC implementation of demand and supply-reduction measures in the GCC countries. A comprehensive assessment for implementing tobacco control measures is needed to adequately understand the tobacco control landscape then evaluate the current efforts to address tobacco use in GCC countries. The purpose of this study was to assess the implementation of national-level FCTC measures in GCC countries during the period of 2008–2020 to define the current state of tobacco control policy efforts in this region.

## Methods

We conducted a longitudinal review of tobacco control measures in the six GCC countries (Bahrain, Kuwait, Oman, Qatar, Saudi Arabia, and UAE) between October 2008 and August 2020. We employed policy surveillance coding methods. We assigned numeric values for answers obtained from the coding questions aimed at exploring tobacco control policies. This method was used to transpose the text of tobacco control policies (qualitative data) into quantitative data to better track and evaluate policies [31].

### Data sources

The WHO FCTC web-based implementation database was used in this study (www.untobaccocontrol.org/impldb/) [32]. The information gathered in this database represents a voluntary submission of countries’ status in implementing FCTC measures. Countries are requested to report biannually to the WHO Convention Secretariat. The raw data extracted from the official country reports formed the WHO FCTC web-based platform. This platform contains only the three most recent country reports (2016, 2018, 2020). In this study, the primary source of data was the official country reports uploaded under country profiles. The total number of official GCC reports included in this study was 31 reports. When country-level reports were unavailable (it may not be uploaded yet in the country profile), the WHO FCTC web-based platform was used.

### Data coding

In the FCTC implementation report, countries are asked to complete a core questionnaire about their progress in implementing FCTC measures. Answers to most questions in the questionnaire are in a narrative format. Two independent reviewers transferred answers from the official country reports to the Microsoft Excel sheet and coded answers to questions using numeric values (i.e., 0/1). Inter-coder reliability was calculated to examine consistency and discrepancy across reviewers (α = 0.98); any disagreement was resolved by discussion between reviewers.

### Descriptive analysis

We examined trends and variations of FCTC measures in GCC countries. Ten key FCTC measures aimed at reducing the demand and supply of tobacco were included in the analysis. Several components related to each FCTC measure were included to explicitly examine the implementation of FCTC in GCC countries. Frequencies of implementing FCTC measures and their components among the countries were analyzed to define the tobacco control landscape in the GCC countries.

## Results

**Fig. 3:**
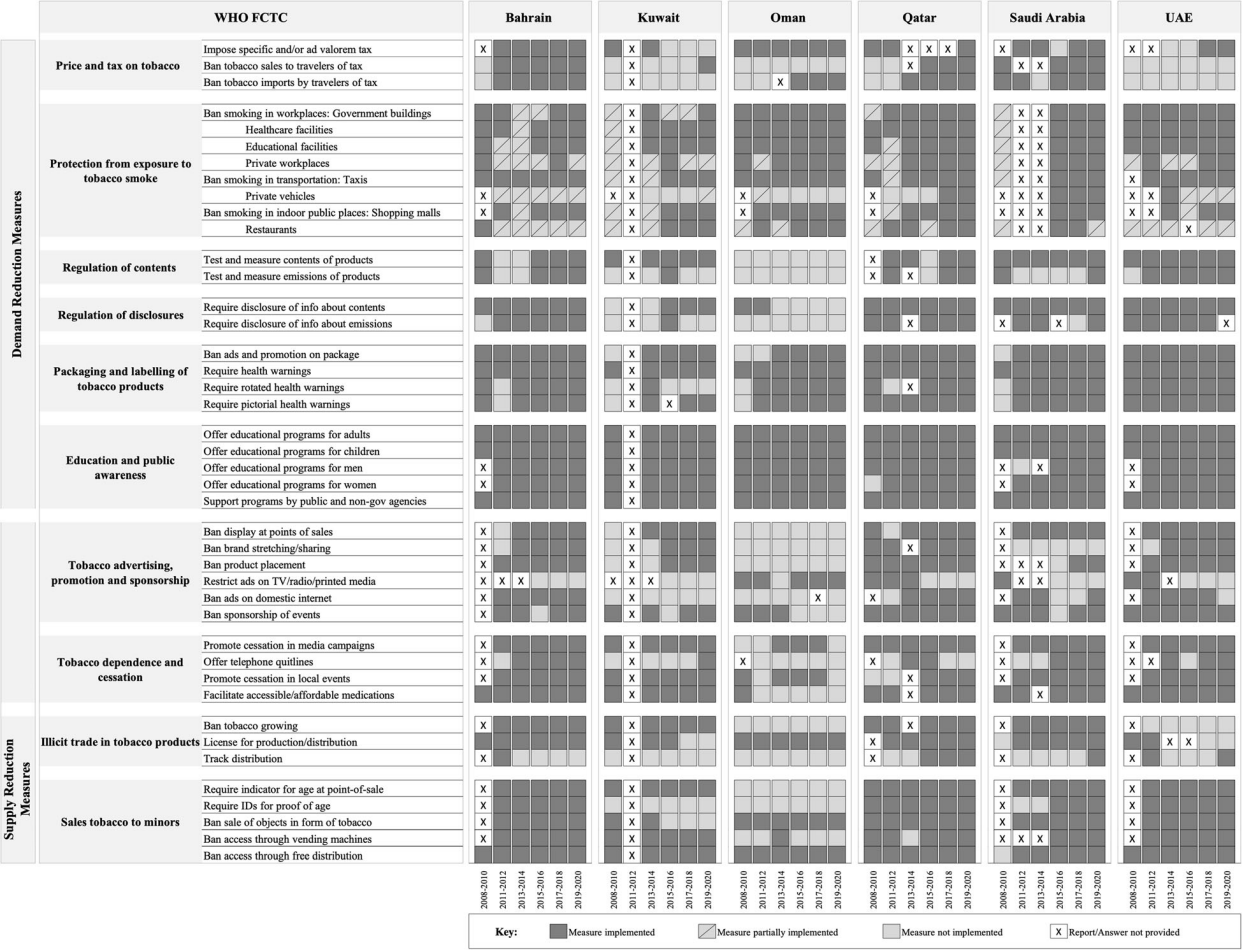
Demonstrates the progress of implementing key FCTC measures, focusing on the demand for and supply of tobacco in GCC countries

As of 2020, the six GCC countries implemented an average 80 % of the ten key FCTC measures targeting the demand for and supply of tobacco, with variation among the individual countries. The implementation of FCTC measures in GCC countries over time is plotted in Fig. 3. Bahrain and Qatar were more comprehensive in implementing FCTC measures compared to the other GCC countries. Kuwait and Oman implemented the least number of FCTC measures. All GCC countries measure tobacco contents, require tobacco importers and manufacturers to disclose to governmental bodies information about products’ contents, and implement essential tobacco packaging and labeling requirements. All GCC countries also provide public awareness programs and celebrate events such as World No Tobacco Day or National No Smoking Day to promote cessation and increase awareness about tobacco’s harmful effects. As of 2020, all countries, except Oman and Qatar, provide a national quitline to facilitate smoking cessation services. All GCC countries, except Oman, offer accessible and affordable medications to treat tobacco dependence.

### Tobacco tax

In GCC countries, tobacco taxes take several forms. The taxes are levied on a specific tax (based on the quantity of products) or/and ad valorem tax (based on the value of products). GCC countries have imposed at least one form of taxes on tobacco products. Only Kuwait has not applied either a specific tax or ad valorem tax on tobacco products since 2016. Four GCC countries have prohibited either or both the imports and sales of tax- and duty-free tobacco products. Kuwait has never banned taxes on tobacco imported by travelers while Oman reported no ban on sales to travelers of tax and duty-free tobacco products. In 2018, the UAE implemented taxes on tobacco products for the first time, but no other price and tax measures.

### Exposure to secondhand smoking

Regulating exposure to smoking in public places is classified as: (a) complete ban where smoking is not allowed under any circumstances; (b) partial ban where smoking is allowed in designated areas; and (c) no ban where no regulation is in place to control exposure to tobacco smoke. GCC countries have started to regulate SHS exposure in public places by implementing a partial ban on smoking in 2008 then shifted to a complete ban in 2020. All countries have entirely banned smoking in public workplaces, shopping malls, and motor vehicles used as places of work like taxis; there is a partial smoking ban in private workplaces in Bahrain and Kuwait. Furthermore, four countries, including Bahrain, Kuwait, Oman, and UAE, have either a partial ban or no regulations to prevent exposure to smoking in private vehicles. Smoking in restaurants is partially banned in Bahrain, Saudi Arabia, and the UAE. Bahrain and Saudi Arabia previously had a complete smoking ban in restaurants, but the UAE always has partially banned smoking in restaurants.

### Tobacco marketing

Most GCC countries, mainly Bahrain, Kuwait, Qatar, and UAE, prohibit tobacco marketing activities, including display ads at point-of-sale, product placement, brand stretching, and tobacco industry sponsorship of events. Oman and Saudi Arabia have never had regulations to prevent tobacco brand stretching and sharing in which tobacco brands are associated with non-tobacco products or services. Interestingly, as of 2016, Bahrain, Kuwait, Oman, and Saudi Arabia had no ban prohibiting the tobacco industry from sponsoring events. Only Oman has restrictions on tobacco advertisements in different media forms such as radio, television, and printed media; Qatar, Saudi Arabia, and the UAE previously had restrictions on media platforms but removed them. Kuwait and Oman have never restricted tobacco advertisements on the domestic internet.

### Tobacco cultivation and trade

At the supply-side of tobacco in GCC countries, only Oman and UAE allow tobacco cultivation in their lands. GCC countries, except Kuwait and UAE, have implemented licensure requirements for tobacco production and distribution to prevent illicit trade. In 2012, Bahrain and UAE had a tracking regime to secure the distribution of tobacco products and facilitate the investigation of the illicit tobacco trade; then, the two countries stopped reporting. As of 2020, only Qatar, Saudi Arabia, and UAE reported having a tracking system. GCC countries require tobacco retail stores to post signage stating the legal age of purchasing tobacco (minimum age ≥ 18). However, Kuwait and Oman have never required tobacco sellers to prove age from purchasers in case of doubt. GCC countries, except Oman, ban alternative strategies that increase tobacco supply and facilitate use by minors, such as tobacco vending machines and free distribution of tobacco products.

## Discussion

This study is the first to assess the progress of implementing FCTC measures targeting both the demand for and supply of tobacco between 2008 and 2020 in GCC countries. It compares the implementation of FCTC measures across GCC countries to identify opportunities and challenges related to tobacco control solutions in the region and provide suggestions to address tobacco use. The current study found improvements in implementing FCTC measures in GCC countries over time. For instance, FCTC measures –offering education and awareness programs, protecting people from SHS, regulating tobacco packaging and labeling, and prohibiting sales of tobacco to minors – have been highly implemented in GCC countries. Yet, the study shows an uneven implementation of FCTC measures across GCC countries. It also reveals an inadequate implementation of certain FCTC measures like increasing tobacco prices and eliminating the illicit tobacco trade.

Unlike this study, several previous studies conducted in GCC countries [26, 27, 33, 34] focused more on the implementation of the MPOWER package that includes measures targeting the demand-side of tobacco such as raising tobacco taxes, enforcing smoke-free, offering cessation for those who want to quit, educating people about the harmful use of tobacco, and banning tobacco marketing activities. Although these studies reported the advancement of implementing FCTC measures in GCC countries, none of them have assessed the implementation of supply-reduction measures [26, 27, 33, 34]. Due to the little support given by global consensus toward policies targeting the supply of tobacco, there is a scant amount of literature focusing on supply-reduction measures and their effectiveness on tobacco use compared to demand-reduction measures [17, 35].

The progress of implementing FCTC measures found in the six GCC countries during the previous decade is consistent with a recent review by Chung-Hall et al. who found rapid progress globally in implementing FCTC measures like bans on smoking in workplaces, public awareness campaigns, and restricting tobacco sales to minors [17]. As mentioned in a previous study, the advancement of implementing FCTC measures in GCC countries could be attributed to the government support to reduce the burden of the tobacco use [36]. Also, the FCTC implementation received support from the public in GCC countries; more than 50 % of teachers, health professionals, and youth in GCC countries support several FCTC measures such as ban smoking in public places, restrict the sale of tobacco to minors, and increase tobacco prices [12].

Although the FCTC treaty helped in diffusing the global tobacco control norms to the national-level measures [37], our results revealed variations in implementing FCTC measures in GCC countries. Consistent with our findings, Heydari et al. reported differences in implementing FCTC measures, mainly MPOWER measures, in the Eastern Mediterranean Region overall [34]. The variations in implementing FCTC measures across GCC countries may reflect the prevalence of tobacco use in each country. For instance, Oman has the lowest rates of tobacco use and implemented the fewest number of FCTC measures while Bahrain has the highest prevalence and implemented the greatest number of FCTC measures [38, 39]. It is also possible that some of the FCTC measures were not stated in the national tobacco control laws or even if there were stated in the books, they might not be effectively enforced. Thus, the variations in the implementation of FCTC measures may be influenced by the level of enforcement of the country-specific tobacco control laws.

The results of this study showed that the evolution of implementing tobacco taxes is slow in GCC countries [28]. Similarly, previous studies indicated that the average price of tobacco products in the Eastern Mediterranean region, including the GCC region, is lower than other regions like Africa, America, South-East Asia, Europe, and Western Pacific [40, 41]. As of 2018, the most sold cigarette brand prices in GCC countries ranged from $2.75/pack in Qatar to $7.33/pack in Saudi Arabia [42]. This finding suggests that lower tobacco prices in one of the GCC countries may impact tobacco sales and consumption in other countries because cross-border cigarette purchasing is a common phenomenon worldwide [43, 44]. In the European region, 24 and 12 % of smokers in France and Germany, respectively, reported purchasing tobacco from cross-border countries like Spain, Luxembourg, and Poland, where tobacco products are cheaper [43]. This possibly occurs in the Arab Gulf region because GCC residents easily cross borders into other countries. Thus, they can purchase duty-free tobacco products from Oman and UAE or cheaper products from Qatar and Kuwait.

The in-depth inventory of tobacco control measures in this study revealed that banning smoking in several public places differs across GCC countries over time, depending on the types of the ban on smoking (complete vs. partial ban). Data indicates that smoking in residential homes and public places (indoor designated areas) is a common and culturally acceptable practice in GCC countries [45]. The widespread phenomenon of smoking in public places such as cafes and restaurants reflects the social acceptability of smoking behavior in the region [46–48]. This social context creates normative beliefs and social pressures to initiate and continue tobacco use [49, 50]; consequently, it undermines the implementation of tobacco control measures [51, 52]. Complete smoking bans in vehicles and public places where people gathered in their leisure time such as cafes, restaurants, and nightclubs still require more effort.

This study reflects the influence of tobacco industries. It showed that tobacco advertising, promotion, and sponsorship are not completely banned in the GCC. The tobacco industry has used several tactics in GCC, ranging from lobbying officials to undermining imposed taxes on cigarettes to marketing tobacco through media and sponsored events [53–55]. The literature indicates that tobacco companies were lobbying against the health warning labels during the 1980 s [56]; however, the results showed that GCC countries implemented all health warning requirements on tobacco packages. Our findings also suggest that the advancement of eliminating illicit tobacco trade has slowly stared in GCC countries. The Middle East Tobacco Association in Jebel Ali, a free trade zone within the UAE, may influence the supply-reduction measures on tobacco. Jebel Ali was previously documented as the main transit for illicit tobacco supply in the Middle East, Europe, and Africa [57]. Tobacco companies have involved in the illicit tobacco trade because smuggled tobacco products are often tax-free and sold in unlicensed stores to vulnerable populations [58]. Knowing that the largest transnational tobacco companies have more than 50 % of the market share in GCC countries would raise a concern about the industry’s interference to challenge tobacco control policies in the region [59].

### Limitations

This study has several limitations. First, it focused on implementing key FCTC measures reported by GCC countries at the national level. This approach may not capture the implementation, not just adoption, of tobacco control measures at the local level within GCC countries; previous literature suggests that tobacco control efforts often start local and percolate to higher jurisdictions [60–62]. Second, this study did not strictly follow the policy surveillance methods. In a typical policy surveillance method, two steps are followed: [1] data collection, key terms are searched in legal database to find legal measures; [2] data coding, numeric values are assigned to legal variables [63]. Our study only applied the data coding step because we were unable to find accessible sources of the laws for each of the GCC countries. We started with the WHO FCTC database, where legal variables related to tobacco control have already been collected through official country-level reporting to the WHO. Finally, this study relied on voluntary country-level reporting which may introduce some inaccuracies or gaps in data. Information submitted via in-country reports might be subject to errors and/or subjective interpretation by officials.

## Conclusions

The comprehensive implementation of FCTC measures has been proven to reduce both the demand and supply of tobacco as these measures were designed to influence both the environment and individual behaviors [18]. Yet, effectively addressing tobacco use will continue to need political commitment at the international, national, and local levels. It is now time for GCC countries to address the gap and share lessons learned in implementing FCTC measures with other countries. Given past influence from the tobacco industry,[53, 54] it is vital to monitor the proliferation and repeal of tobacco control measures at the national and local level. The development of a policy surveillance system can serve as the foundation for ongoing evaluation opportunities in GCC countries. Conducting national-based surveys to monitor tobacco use would also help evaluate tobacco control measures to ensure that policies are working as intended and there are no unintended consequences of the policies. Future studies should focus on policy surveillance and the effect of implementing FCTC measures on the prevalence of tobacco use in GCC countries to allow policymakers to prioritize resources and guide them to implement new interventions.

## Data Availability

The dataset used in this study is available in the WHO FCTC repository. The WHO FCTC web-based implementation database can be accessed through the following link (www.untobaccocontrol.org/impldb/).

## Abbreviations

FCTC: Framework Convention on Tobacco Control
GCC: Gulf Cooperation Council
SHS: Secondhand smoking
UAE: United Arab Emirates
WHO: World Health Organization
